# Topotactic fabrication of transition metal dichalcogenide superconducting nanocircuits

**DOI:** 10.1038/s41467-023-39997-y

**Published:** 2023-07-18

**Authors:** Xiaohan Wang, Hao Wang, Liang Ma, Labao Zhang, Zhuolin Yang, Daxing Dong, Xi Chen, Haochen Li, Yanqiu Guan, Biao Zhang, Qi Chen, Lili Shi, Hui Li, Zhi Qin, Xuecou Tu, Lijian Zhang, Xiaoqing Jia, Jian Chen, Lin Kang, Peiheng Wu

**Affiliations:** 1 Research Institute of Superconductor Electronics, School of Electronic Science and Engineering, College of Engineering and Applied Science, Nanjing University, Nanjing, 210023 China; 2 Hefei National Laboratory, Hefei, 230088 China; 3 Department of Applied Physics, Nanjing University of Aeronautics and Astronautics, Nanjing, 210016 China; 4 Department of Physics, Tsinghua University, Beijing, 100084 China

**Keywords:** Superconducting properties and materials, Synthesis and processing, Superconducting devices

## Abstract

Superconducting nanocircuits, which are usually fabricated from superconductor films, are the core of superconducting electronic devices. While emerging transition-metal dichalcogenide superconductors (TMDSCs) with exotic properties show promise for exploiting new superconducting mechanisms and applications, their environmental instability leads to a substantial challenge for the nondestructive preparation of TMDSC nanocircuits. Here, we report a universal strategy to fabricate TMDSC nanopatterns via a topotactic conversion method using prepatterned metals as precursors. Typically, robust NbSe_2_ meandering nanowires can be controllably manufactured on a wafer scale, by which a superconducting nanowire circuit is principally demonstrated toward potential single photon detection. Moreover, versatile superconducting nanocircuits, *e.g*., periodical circle/triangle hole arrays and spiral nanowires, can be prepared with selected TMD materials (NbS_2_, TiSe_2_, or MoTe_2_). This work provides a generic approach for fabricating nondestructive TMDSC nanocircuits with precise control, which paves the way for the application of TMDSCs in future electronics.

## Abstract (web summary)

The practical device application of transition-metal dichalcogenide superconductors (TMDSCs) is limited by their environmental instability. Here, the authors report a generic, non-destructive, and scalable strategy to fabricate TMDSC nanocircuits via the topotactic conversion of prepatterned metallic precursors.

## Introduction

Since the first discovery of intrinsic superconductivity in exfoliated 2H-NbSe_2_ in the 1970s^1^, 2D transition metal dichalcogenide superconductors (TMDSCs) have drawn considerable research interest and expedited insight into novel physical properties^2–9^. Van der Waals heterostructures based on TMDSC mono/few layers have also spawned exotic superconducting phenomena and new physical mechanisms^10–13^. These novel physical phenomena have also derived many superconducting electronic devices, such as photodetectors^14^, nonreciprocal antennas^15^, and supercurrent diodes^16^. Nanopatterning fabrication is the critical gap in the development of TMDSCs from fundamental research to practical applications^16–20^. Notably, recent advances in fundamental studies on TMDSCs are predominantly conducted on mechanically exfoliated flakes and/or restacked heterostructures, which are still far from practical device applications due to their limited size (<100 μm) and suboptimal stability^3,5,21^.

Recently, large-area TMDSCs have been intensely pursued through a variety of progressive approaches, including chemical intercalation and exfoliation^22^, chemical vapor deposition (CVD)^23,24^, and molecular beam epitaxy (MBE)^25,26^. Strikingly, Lin et al.^24^ reported a two-step growth of environmentally stable wafer-scale TMDSC films, laying the foundation for the future development of integrated superconducting electronic devices. Superconducting nanocircuits patterned from films play an indispensable role in the function and performance of superconducting electronic devices^14,27–33^. For example, in superconducting nanowire single-photon detectors (SNSPDs), ultrathin superconducting films need to be etched into meandered nanowires for ultrahigh detection sensitivity^34,35^. In addition, patterned superconducting systems have induced many exotic physics, e.g., bosonic metallicity^36^, and metallic TMDSC gratings^37^ have been demonstrated to be promising candidates for the near-infrared range. However, despite inspiring achievements, the nondestructive patterning of superconducting nanocircuits on TMDSC films has not been well demonstrated. The multi-step patterning process, involving electron beam lithography and reactive ion etching, inevitably destroys the environmentally unstable TMDSC films. Thus, to advance practical applications in integrated electronics, the development of nondestructive processing of TMDSC nanocircuits is critical but challenging.

Here, we devise a topotactic conversion approach to achieve the nondestructive fabrication of various TMDSC nanocircuits through the chalcogenization of prepatterned transition metals. Typically, multifarious structures (e.g., meandering/spiral nanowires and periodical holey arrays) are patterned on transition metals by electron beam lithography and reaction ion etching, which topotactically transform into targeted TMDSC nanocircuits after annealing in chalcogen atmospheres. A set of microscopies, spectroscopic, and electrical characterizations reveal that the topotactically fabricated NbSe_2_ nanowires retain better structural and superconducting properties compared to the counterpart obtained from patterning NbSe_2_ films. A demoed device on a NbSe_2_ circuit delivers regular critical currents in different widths under different temperatures, demonstrating its high quality and potential for further application in integrated electronics.

## Results

### Strategies for preparing TMDSC nanocircuits

**Fig. 1:**
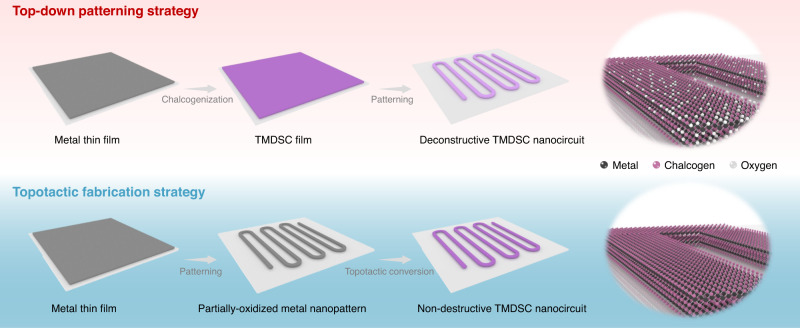
Schematic illustrations of top-down patterning and topotactic fabrication strategies for preparing transition metal dichalcogenide superconductors (TMDSC) nanocircuits. The top-down patterning method involves three steps: (i) deposition of metal films on a substrate; (ii) chalcogenization of metal films into TMDSC films; and (iii) etching of TMDSC films into nanopatterns with severe degradation. In contrast, the topotactic fabrication method topotactically chalcogenizes the prefabricated partially-oxidized metal nanopatterns, which avoids the destruction of the TMDSCs from the patterning process.

TMDSCs have many novel phenomena in quantum physics and show potential in both fundamental research and practical applications, for which the fabrication of TMDSC nanocircuits is essential. The traditional top-down patterning strategy that patterns a film into the targeted nanocircuit has been widely applied in the electronic community. However, this process involves a set of chemical treatments, including resist coating, baking, developing, and lift-off in organics, which inevitably destroy the environmentally unstable TMDSCs. As illustrated in Fig. 1, a TMDSC film is first synthesized by topotactic conversion of the metal film sputtered on a substrate^38^. Then, the as-prepared film is top-down patterned into specific nanocircuits, e.g., meandered nanowires. This process could result in the generation of oxidized species and vacancies in the TMDSC nanocircuit, which largely deteriorate its structural and superconducting properties. To meet this challenge, a nondestructive topotactic fabrication method is developed. In detail, the metal film is first patterned into selected nanostructures and then chalcogenized into a nanostructured TMDSC through a topotactic conversion procedure. Impressively, all the patterning processes are applied forward to the metal precursors rather than TMDSCs; as a result, nondestructive fabrication of TMDSC nanocircuits can be readily realized.

### Characterization of NbSe_2_ meandered nanowire

**Fig. 2:**
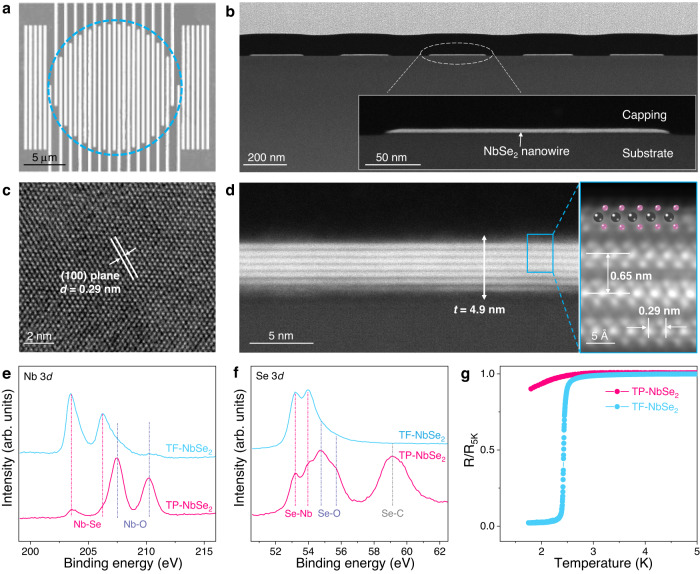
Characterization of NbSe_2_ meandered nanowire prepared by topotactic fabrication strategy (TF-NbSe_2_). **a** Scanning electron microscopy (SEM) image of TF-NbSe_2_, in which a gray meandered nanowire on a white background is highlighted by a dashed blue circle. **b** Cross-sectional scanning transmission electron microscopy (STEM) image of TF-NbSe_2_ nanowire and inset is the enlarged image of a single TF-NbSe_2_ nanowire. **c** In-plane high-resolution transmission electron microscopy (HRTEM) image of TF-NbSe_2_ with a lattice distance (*d*) of 0.29 nm for (100) plane. **d** High-resolution STEM image of TF-NbSe_2_ along with enlarged atomic-resolution display, showing the van der Waals layered structure with a thickness (*t*) of 4.9 nm and a layer distance of 0.65 nm. The Nb and Se atoms are represented with black and pink spheres, respectively. Core-level Nb 3*d* (**e**) and Se 3*d* (**f**) X-ray photoemission spectroscopy (XPS) spectra of TF-NbSe_2_ and NbSe_2_ prepared by top-down patterning strategy (TP-NbSe_2_). The vertical dash lines refer to the bonds below in the same color. **g** Temperature dependence of the resistance for TF-NbSe_2_ and TP-NbSe_2_. R_5K_ means the resistance at the temperature of 5 K.

As a prototypical TMDSC, *2H*-NbSe_2_ has attracted much attention due to its unique out-of-plane or Ising spin-orbit copling^4^. Here, the nondestructive fabrication of a NbSe_2_ meandered nanowire is demonstrated by the proposed topotactic fabrication strategy (defined as TF-NbSe_2_). First, a dense Nb film with a thickness of 2 nm (roughness of 0.2 nm) was deposited on the sapphire substrate by magnetron sputtering (Supplementary Fig. 1a). The 2 nm thick Nb film was metallic, and the resistance decreased as the temperature decreased; however, it cannot exhibit superconducting properties at a temperature of approximately 1.8 K, which should be attributed to surface oxidation (Supplementary Fig. 1b)^39^. The X-ray photoemission spectroscopy (XPS) and time-of-flight secondary ion mass spectrometry results confirm that Nb was partially oxidized into a composite of Nb and Nb_2_O_5_ after patterning by electron beam lithography and reaction ion etching; consequently, a partially oxidized Nb meandered nanowire with a width of 200 ± 10 nm was prepared (Supplementary Fig. 1c–f). The partially oxidized Nb meandered nanowire was transformed into a NbSe_2_ meandered nanowire by topotactic selenization. In the Raman spectrum (Supplementary Fig. 2a), two peaks at 229 and 243 cm^−1^ can be assigned to the A_1g_ and E^1^_2g_ modes of NbSe_2_, respectively, indicating the topotactic transformation of the Nb precursor into NbSe_2_^19,40^. The scanning electron microscopy (SEM) and atomic force microscopy (AFM) images (Fig. 2a and Supplementary Fig. 2b–d) show that a continuous meandered NbSe_2_ nanowire is obtained, which has a dense surface and a uniform linewidth. Note that the height of the NbSe_2_ nanowire is determined to be ~5 nm, which is 2.2-fold that of the partially-oxidized Nb precursor (2.3 nm). The wafer photography and XRD results demonstrate that the prepatterned Nb precursor is fully and macroscopically transformed into NbSe_2_ (Supplementary Fig. 3). Furthermore, the thickness and width of the NbSe_2_ nanowires can be precisely controlled by tuning the sputtering time and electron beam lithography parameters (Supplementary Fig. 4).

Aberration-corrected scanning transmission electron microscopy (STEM) was used to reveal the microstructure of NbSe_2_. As shown in Fig. 2b, the cross-sectional image of the meandered NbSe_2_ nanowire shows a uniform width of 200 nm, and the enlarged image shows the continuity and homogeneity of the TF-NbSe_2_. The in-plane high-resolution transmission electron microscopy (HRTEM) image (Fig. 2c) reveals the in-plane atomic arrangement of NbSe_2_, in which the lattice distances of 0.29 nm corresponds to the (100) plane. The high-resolution STEM image of TF-NbSe_2_ (Fig. 2d) shows the layered structure and typical atomic arrangement of the NbSe_2_ crystal without any oxidized species. The layer-by-layer van der Waals structure of NbSe_2_, with a layer thickness of 0.65 nm and a total thickness of 4.9 nm^25,41^, is in agreement with the AFM result. Even at the edge of the TF-NbSe_2_ nanowire, the layered structure is clearly observed without distinguishable oxidation (Supplementary Fig. 5). The corresponding EDS mappings further confirm the intact NbSe_2_ composition at the edge of TF-NbSe_2_ nanowire with a negligible oxygen signal. For comparison, another meandered NbSe_2_ nanowire is prepared by etching a NbSe_2_ film, which is derived from traditional top-down patterning (defined as TP-NbSe_2_) (Supplementary Fig. 6).

While the two kinds of meandered NbSe_2_ nanowires exhibit similar morphologies, their intrinsic properties vary extremely. First, the chemical state and composition of the two samples are examined by XPS. As shown in Fig. 2e, two pairs of peaks at 203.4/206.2 and 207.5/210.3 eV are observed in the Nb 3*d* XPS spectrum of the TF-NbSe_2_ sample, which is attributed to Nb^4+^ (NbSe_2_) and Nb^5+^ (Nb_2_O_5_), respectively^42^. It should be noted that the slight oxidation in TF-NbSe_2_ arises from surface oxidation during XPS measurement. In contrast, the peaks referring to Nb_2_O_5_ species are in the majority of the Nb 3*d* XPS spectrum for the TP-NbSe_2_ sample. This suggests that TP-NbSe_2_ experiences severe oxidation during the patterning process. Based on the area of the signal peak after deconvolution (Supplementary Fig. 7), TF-NbSe_2_ is determined to be composed of 93.67% NbSe_2_ and 6.33% Nb_2_O_5_, while TP-NbSe_2_ contains 26.7% NbSe_2_ and 73.3% Nb_2_O_5_. The Nb/Se/O ratios are calculated to be 1/1.76/0.30 and 1/0.31/2.12 for TF-NbSe_2_ and TP-NbSe_2_, respectively (Supplementary Table 1). In addition, two pairs of peaks at 53.3/54.0 and 54.6/55.3 eV, corresponding to Se^2-^ (NbSe_2_) and Se^4+^ (SeO_2_), respectively, are present in the Se 3*d* XPS spectra of both TF-NbSe_2_ and TP-NbSe_2_ (Fig. 2f)^43^. Obviously, the proportion of SeO_2_ in TP-NbSe_2_ is much higher than that in TF-NbSe_2_, indicating even more serious oxidation of NbSe_2_, which is consistent with Nb 3*d* XPS analyses. Notably, one more peak at 58.9 eV attributed to the C–Se bond emerges in TP-NbSe_2_^44^. It is speculated that the organic resist applied in the patterning process can react with NbSe_2_ and form C–Se species, which will undoubtedly deteriorate the quality of the NbSe_2_ nanocircuit. Notably, TF-NbSe_2_ shows competitive quality in structure and composition to NbSe_2_ film prepared by selenization of Nb film (Supplementary Fig. 8), indicating that partial oxidation of Nb nanopattern hardly affects the TF-NbSe_2_ quality.

To further characterize the superconducting quality, the transport properties at low temperatures of TP-NbSe_2_ and TF-NbSe_2_ are compared. Figure 2g shows the temperature dependence of the normalized longitudinal resistance at zero magnetic field. For TF-NbSe_2_, its resistance begins to plunge at ~2.7 K and reaches zero at ~2.2 K, suggesting the trigger of superconductivity. In contrast, the resistance of TP-NbSe_2_ only slightly decreases as the temperature decreases from 2.9 to 1.8 K, corresponding to a nonsuperconducting state. A TP-NbSe_2_ nanowire reported by Mills et al. exhibited a 31.4% decrease in *T*_c_ compared to the initial NbSe_2_ flake, indicating a substantial degradation in the superconductivity caused by the destructive patterning process^19^. Obviously, the NbSe_2_ nanopatterns obtained by the topotactic fabrication strategy can retain excellent superconductivity compared to that from top-down patterning.

### Demonstration of TF-NbSe_2_ nanowire devices

**Fig. 3:**
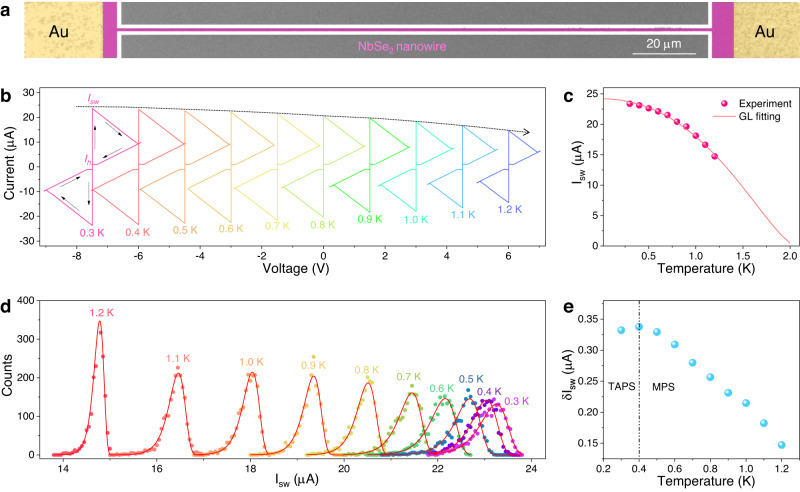
Superconducting properties of TF-NbSe_2_ nanowire device. **a** SEM image of a 200-nm-wide NbSe_2_ nanowire. **b** *I–V* curves of 200-nm-wide NbSe_2_ device under different temperatures. The curves are horizontally offset for clarity, and the arrows represent the bias current (*I*_*b*_) scan order. **c** The average values of switch current (*I*_*sw*_) for 200-nm-wide NbSe_2_ device under different temperatures, which can be fitted by the Ginzburg–Landau model. **d** Distribution of *I*_*sw*_ values for 200-nm-wide NbSe_2_ device at different temperatures, which follows Gumbel fitting. **e** Standard deviation *I*_*sw*_ (δ*I*_*sw*_*)* of 200-nm-wide NbSe_2_ device under different temperatures, in which 400 mK is the crossover temperature of thermally active phased-slip (TAPS) and multiple phase-slip (MPS).

To investigate the feasibility of the topotactic fabrication strategy, a superconducting device based on TF-NbSe_2_ nanowires is demoed. Figure 3a displays the SEM image of the 200-nm-wide NbSe_2_ nanowire device with one electrode side connected to the ground electrode (GND), which is systematically evaluated by current–voltage (*I–V*) characteristics at low temperatures. A measuring circuit is needed to conduct the electrical measurements. As shown in Supplementary Fig. 9, the adjustable voltage source meter (*V*_*S*_) series connection with resistor R_0_ (100 kΩ) provides variable currents to the devices; thus, the bias current (*I*_*b*_) is input to the DC terminal of the bias-tee and then to the device^45^. Figure 3b displays the *I–V* curves of the 200-nm-wide NbSe_2_ device at different temperatures with a current sweep rate of 0.2 µA s^−1^. Clearly, the device remains in the superconducting state until the *I*_*b*_ exceeds the switch current (*I*_*sw*_, where the superconducting device transitions to a nonsuperconducting state). Once *I*_*b*_ >*I*_*sw*_, the device immediately transforms to a normal state and generates resistance. With decreasing *I*_*b*_ below *I*_*sw*_, the device begins with a normal state due to residual Joule heating and finally recovers to the superconducting state at the hysteresis current (*I*_*h*_). Among them, the *I*_*sw*_ value, relative to the number of Cooper pairs, increases with decreasing operation temperature, and the *I*_*h*_, immune to noise, reflects the equilibrium state between Joule heat and heat dissipation in the nanowire^46^. The *I*_*sw*_/*I*_*h*_ is determined to be over 21 at 1 K (Supplementary Table 2), which is higher than those reported for superconducting nanowires^47^, suggesting promise for application in superconducting single-photon detectors. The relationship between *I*_*sw*_ mean values and operation temperature is plotted in Fig. 3c, which can be fitted according to the Ginzburg–Landau (GL) theory^48^. The good agreement between the experimental data and the fittings suggests that the superconductivity of the 200-nm-wide NbSe_2_ nanowire basically obeys GL theory, and the *T*_*c*_ of the 200-nm-wide NbSe_2_ nanowire is determined to be approximately 2 K by fitting the GL theory.

Furthermore, phase fluctuations reveal the intrinsic fluctuation of the superconductors and are the cause of the superconducting transition, representing the stability of the system under certain temperatures, that is, the distribution of *I*_*sw*_ when the superconductor switches to the normal state. Here, we further performed 2000 *I–V* sweeps with a sweep rate of 5 nA s^−1^ to record the distribution of *I*_*sw*_ values of the 200-nm-wide NbSe_2_ device at different operation temperatures (Supplementary Fig. 10). After frequency counting with a bin size of 50 nA, all the *I*_*sw*_ distributions are basically consistent with the Gumbel fitting from 300 mK to 1.2 K (Fig. 3d)^49^. The standard deviation *δI*_*sw*_ of each distribution under a certain temperature is calculated and plotted in Fig. 3e. The overall trend follows the conventional phase-slip theory, and *δI*_*sw*_ reaches a peak at 400 mK, which decreases with the operating temperature from 400 mK to 1.2 K and slightly decreases after the temperature is below 400 mK. This indicates that 400 mK is the crossover temperature of the thermally active phase-slip (TAPS) and multiple phase-slip (MPS) for the NbSe_2_ device^46,50^. Moreover, NbSe_2_ nanowire devices with different widths of 200, 400, 800, and 1000 nm are fabricated and compared (Supplementary Fig. 11). All the relationships between *I*_*sw*_ and operation temperature for the other three devices are in accordance with GL theory (Supplementary Fig. 12).

Based on the abovementioned analyses, the traditional top-down patterning approach cannot meet the nondestructive fabrication of TMDSC nanocircuits, which usually suffer from heavy degradation during chemical treatments. To this end, a topotactic fabrication strategy is developed, by which nondestructive TMD (with NbSe_2_ as a typical instance) superconducting nanocircuits with tunable parameters (e.g., thickness and width) can be produced through topotactical chalcogenization of prepatterned metal precursors. Comparative characterizations demonstrate the superior structural and superconducting properties of the TF-NbSe_2_ nanocircuit. Moreover, a superconducting device is demoed by TF-NbSe_2_, exhibiting a typical BCS superconductor. It can be concluded that the topotactic fabrication strategy proposed in this work can overcome the bottleneck of the traditional top-down patterning method when facing the fabrication of environmentally unstable TMDSCs.

### The universality of topotactic fabrication strategy

**Fig. 4:**
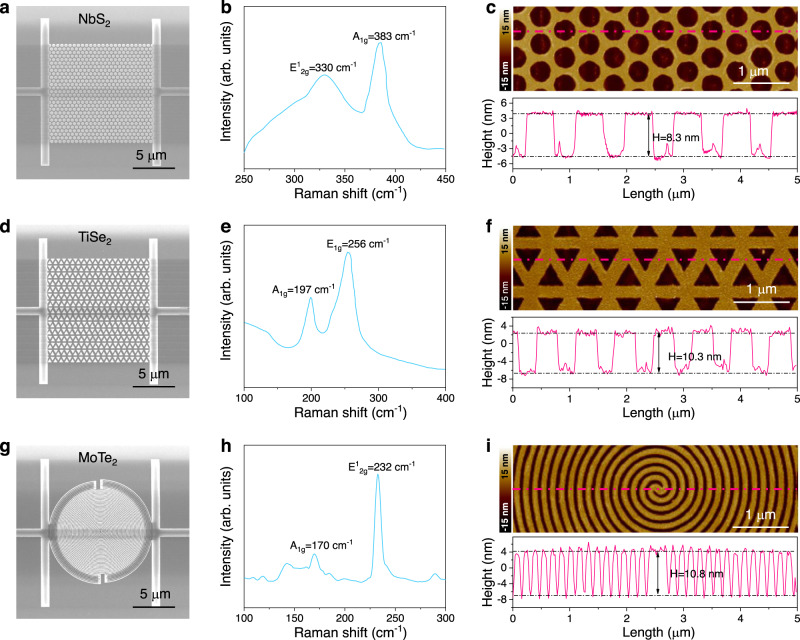
Universality of topotactic fabrication strategy. SEM images, Raman spectra, and AFM images of circle-holey NbS_2_ (**a**–**c**), triangle-holey TiSe_2_ (**d**–**f**), and spiral MoTe_2_ nanowires (**g**–**i**). The height (*H*) profiles in **c**, **f**, and **i** are derived from the dashed red line in relative AFM images.

The topotactic fabrication strategy can prevent TMDSCs from suffering damage during the patterning process and realize the nondestructive fabrication of TMDSC nanocircuits for potential device applications. The meandered NbSe_2_ superconducting nanowire reaches a length of over 200 μm with superior continuity and homogeneity. By this topotactic fabrication strategy, the length of nanocircuits is determined by the prepatterning process but is independent of the TMD materials. Strikingly, this method is widely applicable to the nondestructive preparation of diverse TMDSC nanocircuits in different structures and compositions. For structure, holey patterning of superconductors provides a platform to exploit exotic superconductivity fundamentally, e.g., bosonic metallicity^36^. For material, TiSe_2_ is another representative TMDSC with inspiring superconductivity and charge density waves^51,52^. To prove the universality of the topotactic fabrication strategy, superconducting nanocircuits of circle-holey NbS_2_, triangle-holey TiSe_2_, and spiral MoTe_2_ nanowires are fabricated (Fig. 4 and Supplementary Fig. 13). It is undoubted that this strategy is also applicable to TMD semiconductor nanopatterns for integrated devices (Supplementary Fig. 14). These instances support the promise of the topotactic fabrication strategy in manufacturing high-quality TMDSC nanocircuits for both fundamental research and practical applications.

## Discussion

In summary, we have demonstrated a facile and universal strategy for approaching the nondestructive fabrication of TMDSC nanocircuits, including meandered/spiral nanowires and circle/triangle-holey arrays, based on a topotactic conversion mechanism. The strategy employs topotactic chalcogenization of prepatterned metal precursors, which can effectively avoid damage from the patterning process and retain the superconductivity of the TMDSC nanocircuits (demoed by NbSe_2_ nanowire devices). Notably, this approach enables the preparation of diverse TMDSC nanocircuits with tunable thickness and width. As such, this scalable fabrication technique ensures the nondestructive production of high-quality TMDSC nanocircuits with precise control. It is beyond doubt that with further development and optimization, more kinds of structures (e.g., in-plane/out-of-plane heterostructures) and compositions (e.g., ternary/quaternary alloys) with striking superconducting properties could be accessible, which will promote the practical application of TMDSCs in functional electronics.

## Methods

### Fabrication of NbSe_2_ meandered nanowires

A topotactic fabrication strategy was developed to prepare nondestructive NbSe_2_ meandered nanowires. First, Nb thin films were deposited on single-side polished *c*-plane sapphire substrates (Hefei Kejing) by magnetron sputtering (base pressure <9 × 10^−6^ Pa). During the sputtering process, the working power, working pressure, gas flow rate, and deposition rate were set to 100 W, 3 mTorr, 80 sccm, and 0.2 nm s^−1^, respectively.

Second, the sputtered Nb films were patterned into meandered nanowires. A layer of polymethyl methacrylate (PMMA, Allresist) electron beam resist was spin-coated on the Nb film. After exposure by E-beam lithography (EBL, Raith), the film was developed in methyl isobutyl ketone (MIBK, Allresist) for 90 s and isopropanol (Aladdin, 99%) for 60 s to obtain the desired meandered nanowire structure on PMMA. Then, the exposed film was etched for 40 s by reactive ion etching (RIE, Samco RIE-10NR) with a CF_4_ flow of 40 sccm, a working pressure of 4 Pa, and a working power of 100 W. The residual PMMA was removed through a water bath with N-methyl pyrrolidone (Aladdin, 99%) at 80 °C for 30 min.

Finally, a two-zone furnace was used for the topotactic selenization of the patterned Nb nanowires. Selenium powder (500 mg) and patterned Nb sample were placed at zones I (upstream) and II (downstream), which were heated to 450 and 800 °C, respectively. Ar (50 sccm) and H_2_ (50 sccm) were used as carrier gases. After annealing for 6 min, the furnace was rapidly cooled to room temperature.

For comparison, NbSe_2_ meandered nanowires were also prepared by a top-down patterning method. The as-sputtered Nb films were first selenized into NbSe_2_ films, followed by patterning into meandered nanowires.

The topotactic fabrication strategy can be adapted to generally fabricate various TMDSC nanopatterns, including circle-holey NbS_2_, triangle-holey TiSe_2_, and spiral MoTe_2_ nanowires. The detailed parameters are listed in Supplementary Tables 3 and 4.

### Characterizations

SEM and AFM images were captured by Compact Melin (Zeiss) and Dimension Icon (Bruker), respectively. TEM images were captured by an FEI TECNAI G2 F20 200 kV. Cross-sectional HAADF-STEM images were captured by a Titan Cubed G2 60–300 system. XPS spectra were collected with a Thermo Fisher Es-calab. Raman spectra were obtained on a LabRAM HR Evolution (Horiba) with a 532 nm laser. All samples were fabricated by the EBL system (Raith, EBPG5200) and RIE (Samco RIE-10NR).

### Electrical measurements

The electrical transport characteristics of devices were carried out in the Dilution refrigerating machine for Trition 9 (Oxford Instruments). The measuring circuit includes a digital source meter (keysight 2901) and a T-shaped bias tee. The RF&DC port of the T-shaped Bias-tee is connected to the coaxial cable, leading to devices under extremely low temperatures. The DC port is connected to a digital source meter and the resistor *R*_0_ (100 kΩ) to provide a constant current source, and the RF port is connected to an *R*_1_ (50 Ω) load resistance. Before the measurements, spot welding is used to connect the Ti/Au electrodes to the coaxial cable. The detailed method and corresponding real pictures are presented in Supplementary Fig. 15.

## Supplementary information

Supplementary Information

Peer Review File

## Data Availability

The Source Data underlying the figures of this study are provided in the paper. All raw data generated during the current study are available from the corresponding authors upon request. Source data are provided in this paper.

## Source data

Source Data

## References

[1] Frindt RF (1972). Superconductivity in ultrathin NbSe_2_ layers. Phys. Rev. Lett..

[2] Calandra M, Mauri F (2011). Charge-density wave and superconducting dome in TiSe_2_ from electron-phonon interaction. Phys. Rev. Lett..

[3] Xi XX (2016). Ising pairing in superconducting NbSe_2_ atomic layers. Nat. Phys..

[4] Sohn E (2018). An unusual continuous paramagnetic-limited superconducting phase transition in 2D NbSe_2_. Nat. Mater..

[5] Zheliuk O (2019). Josephson coupled Ising pairing induced in suspended MoS_2_ bilayers by double-side ionic gating. Nat. Nanotechnol..

[6] Liu XL, Chong YX, Sharma R, Davis JCS (2021). Discovery of a Cooper-pair density wave state in a transition-metal dichalcogenide. Science.

[7] Yan D (2020). NbSeTe-a new layered transition metal dichalcogenide superconductor. J. Phys. Condens. Mattter.

[8] Zhang HX (2022). Tailored Ising superconductivity in intercalated bulk NbSe_2_. Nat. Phys..

[9] Xi XX, Berger H, Forro L, Shan J, Mak KF (2016). Gate tuning of electronic phase transitions in two-dimensional NbSe_2_. Phys. Rev. Lett..

[10] Xu JP (2015). Experimental detection of a Majorana mode in the core of a magnetic vortex inside a topological insulator-superconductor Bi_2_Te_3_/NbSe_2_ heterostructure. Phys. Rev. Lett..

[11] Kezilebieke S (2020). Topological superconductivity in a van der Waals heterostructure. Nature.

[12] Zhao WM (2022). Moire enhanced charge density wave state in twisted 1T-TiTe_2_/1T-TiSe_2_ heterostructures. Nat. Mater..

[13] Yokoi M (2020). Negative resistance state in superconducting NbSe2 induced by surface acoustic waves. Sci. Adv..

[14] Walsh ED (2021). Josephson junction infrared single-photon detector. Science.

[15] Zhang EZ (2020). Nonreciprocal superconducting NbSe_2_ antenna. Nat. Commun..

[16] Bauriedl L (2022). Supercurrent diode effect and magnetochiral anisotropy in few-layer NbSe_2_. Nat. Commun..

[17] Jindal A (2023). Coupled ferroelectricity and superconductivity in bilayer T_d_-MoTe_2_. Nature.

[18] de la Barrera SC (2018). Tuning Ising superconductivity with layer and spin-orbit coupling in two-dimensional transition-metal dichalcogenides. Nat. Commun..

[19] Mills SA (2014). Single-crystal superconducting nanowires of NbSe_2_ fabricated by reactive plasma etching. Appl. Phys. Lett..

[20] Wang XH (2023). All-water etching-free electron beam lithography for on-chip nanomaterials. ACS Nano.

[21] Efetov DK (2016). Specular interband Andreev reflections at van der Waals interfaces between graphene and NbSe_2_. Nat. Phys..

[22] Li J (2021). Printable two-dimensional superconducting monolayers. Nat. Mater..

[23] Wang H (2017). High-quality monolayer superconductor NbSe_2_ grown by chemical vapour deposition. Nat. Commun..

[24] Lin HH (2019). Growth of environmentally stable transition metal selenide films. Nat. Mater..

[25] Zhao K (2019). Disorder-induced multifractal superconductivity in monolayer niobium dichalcogenides. Nat. Phys..

[26] Chen P (2015). Charge density wave transition in single-layer titanium diselenide. Nat. Commun..

[27] Arute F (2019). Quantum supremacy using a programmable superconducting processor. Nature.

[28] Wang FF (2022). In vivo non-invasive confocal fluorescence imaging beyond 1,700 nm using superconducting nanowire single-photon detectors. Nat. Nanotechnol..

[29] Gol’tsman GN (2001). Picosecond superconducting single-photon optical detector. Appl. Phys. Lett..

[30] Mazin BA (2006). Position sensitive x-ray spectrophotometer using microwave kinetic inductance detectors. Appl. Phys. Lett..

[31] Fagaly RL (2006). Superconducting quantum interference device instruments and applications. Rev. Sci. Instrum..

[32] Ullom JN, Bennett DA (2015). Review of superconducting transition-edge sensors for X-ray and gamma-ray spectroscopy. Supercond. Sci. Tech..

[33] Shurakov A, Lobanov Y, Goltsman G (2015). Superconducting hot-electron bolometer: from the discovery of hot-electron phenomena to practical applications. Supercond. Sci. Tech..

[34] Zadeh IE (2021). Superconducting nanowire single-photon detectors: a perspective on evolution, state-of-the-art, future developments, and applications. Appl. Phys. Lett..

[35] Guan Y (2022). SNSPD array with single-channel readout based on compressive sensing. ACS Photonics.

[36] Yang C (2022). Signatures of a strange metal in a bosonic system. Nature.

[37] Zhao M (2021). Electrostatically tunable near-infrared plasmonic resonances in solution-processed atomically thin NbSe_2_. Adv. Mater..

[38] Xiao X, Wang H, Urbankowski P, Gogotsi Y (2018). Topochemical synthesis of 2D materials. Chem. Soc. Rev..

[39] Lin HH (2023). Tunability of the superconductivity of NbSe_2_ films grown by two-step vapor deposition. Molecules.

[40] Zhou J (2018). A library of atomically thin metal chalcogenides. Nature.

[41] Chen C (2020). Strain-controlled superconductivity in few-layer NbSe_2_. ACS Appl. Mater. Inter..

[42] Park S (2020). Tailoring domain morphology in monolayer NbSe_2_ and W_x_Nb_1-x_Se_2_ heterostructure. ACS Nano.

[43] Park H, Kim JY, Oh JY, Lee TI (2020). Long-term stable NbSe_2_ nanosheet aqueous ink for printable electronics. Appl. Surf. Sci..

[44] Liu L (2021). A stable and ultrafast K ion storage anode based on phase-engineered MoSe_2_. Chem. Commun..

[45] Gaggero A (2019). Amplitude-multiplexed readout of single photon detectors based on superconducting nanowires. Optica.

[46] Zhang B (2022). Photon-assisted phase slips in superconducting nanowires. Phys. Rev. Appl..

[47] Chen Q (2022). Suppression of superconductivity dominated by proximity effect in amorphous MoSi nanobelts. Phys. Rev. B.

[48] Il’in K, Siegel M, Semenov A, Engel A, Hübers HW (2005). Critical current of Nb and NbN thin‐film structures: the cross‐section dependence. Phys. Status Solidi C.

[49] Li P (2011). Switching currents limited by single phase slips in one-dimensional superconducting Al nanowires. Phys. Rev. Lett..

[50] Puglia C, De Simoni G, Giazotto F (2020). Electrostatic control of phase slips in Ti Josephson nanotransistors. Phys. Rev. Appl..

[51] Joe YI (2014). Emergence of charge density wave domain walls above the superconducting dome in 1T-TiSe_2_. Nat. Phys..

[52] Sun L (2017). Suppression of the charge density wave state in two-dimensional 1T-TiSe_2_ by atmospheric oxidation. Angew. Chem. Int. Ed..

