# Supplementary material for: Topotactic fabrication of transition metal dichalcogenide superconducting nanocircuits
Source: Nat Commun. 2023 Jul 18;14:4282. doi: 10.1038/s41467-023-39997-y (PMC10354018; doi:10.1038/s41467-023-39997-y)
Supplement: Supplementary file 1 — Supplementary Information [file 41467_2023_39997_MOESM1_ESM.pdf]

Supplementary Information

Topotactic fabrication of transition-metal dichalcogenide superconducting nanocircuits

Xiaohan Wang,[1,#] Hao Wang,[1,2#*] Liang Ma,[1,#] Labao Zhang,[1,2*] Zhuolin Yang,[1] Daxing Dong,[3] Xi Chen,[4] Haochen Li,[1] Yanqiu Guan,[1] Biao Zhang,[1] Qi Chen,[1] Lili Shi,[1] Hui Li,[1] Zhi Qin,[1] Xuecou Tu,[1] Lijian Zhang,[1] Xiaoqing Jia,[1,2] Jian Chen,[1] Lin Kang,[1,2] Peiheng Wu[1,2*]

[1] Research Institute of Superconductor Electronics, School of Electronic Science and Engineering, College of Engineering and Applied Science, Nanjing University, Nanjing 210023, China

[2]Hefei National Laboratory, Hefei 230088, China

[3]Department of Applied Physics, Nanjing University of Aeronautics and Astronautics, Nanjing 210016, China

[4]Department of Physics, Tsinghua University, Beijing 100084, China

[#]These authors contributed equally to this work.

*Correspondence to: wanghao91@nju.edu.cn; Lzhang@nju.edu.cn; phwu@nju.edu.cn.

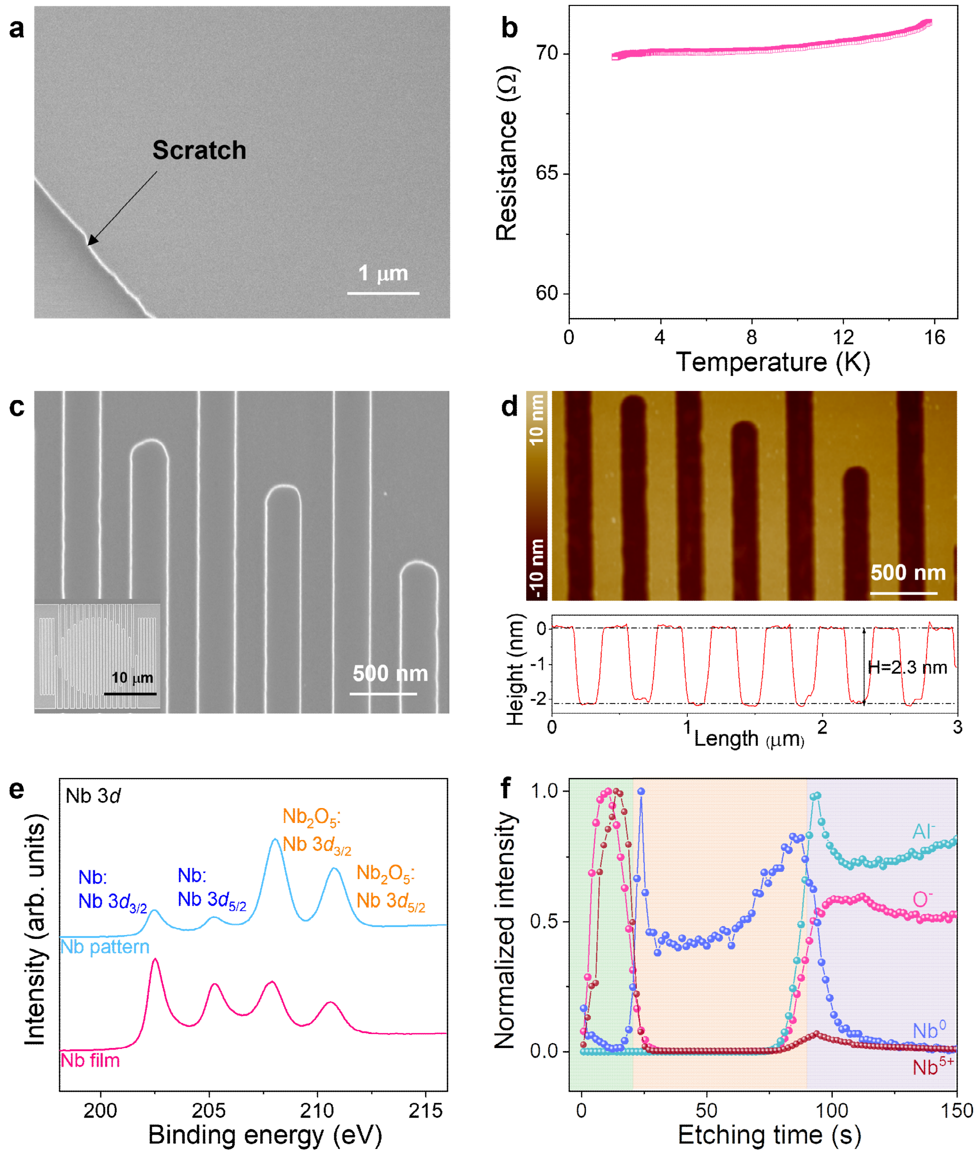

Supplementary Figure 1 Characterization of the Nb film. (a) SEM image of the Nb film. (b) RT curve of a 2 nm thick Nb film. (c) SEM and (d) AFM images of meandered Nb nanowires. (e) Core-level Nb 3*d* spectra of the Nb film before and after patterning. (f) Normalized TOF-SIMS depth profile of the ultrathin Nb film after nanopatterning.

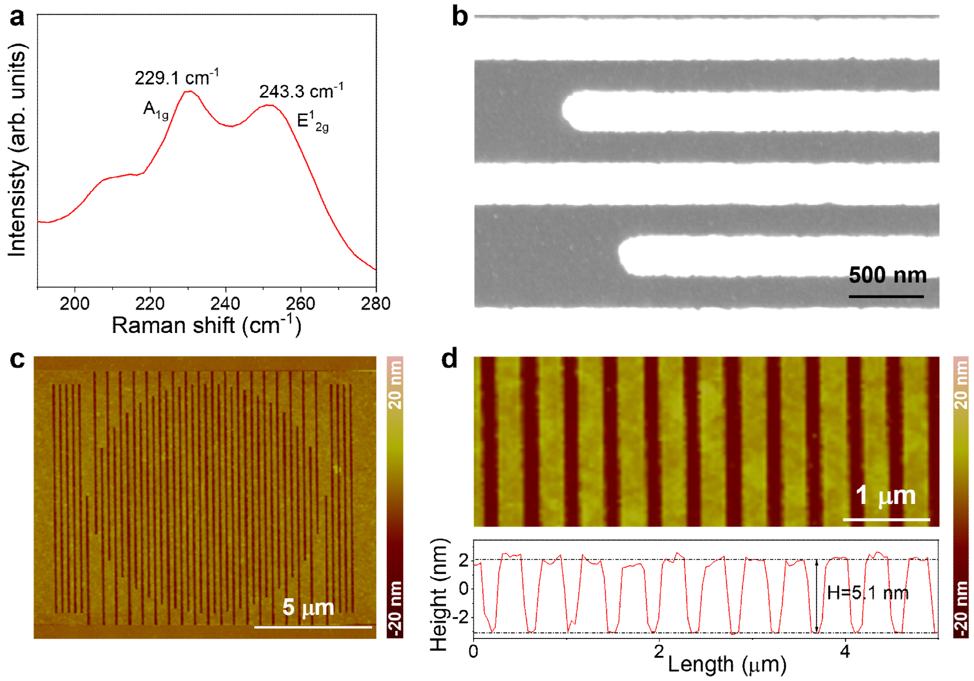

Supplementary Figure 2 Characterization of the TF-$NbSe_2$ meandered nanowires. (a) Raman spectrum, (b) SEM images and (c-d) AFM images of the TF-$NbSe_2$ meandered nanowires.

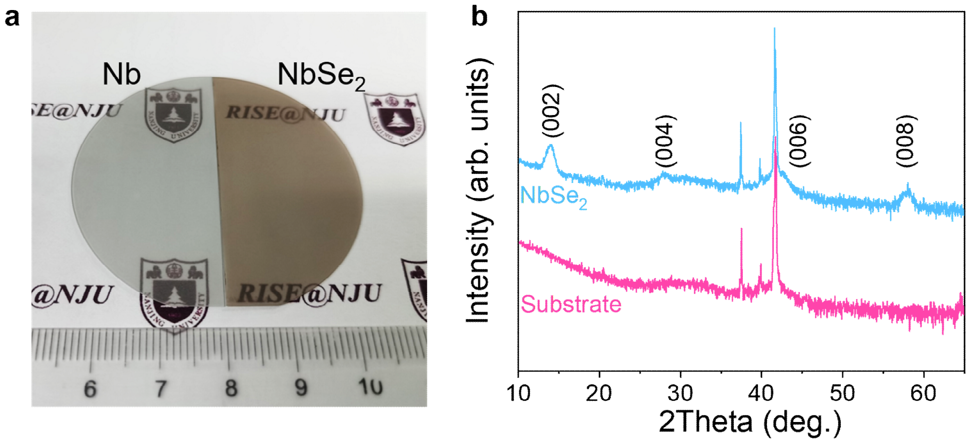

Supplementary Figure 3 (a) Macroscopic photography of TF-$NbSe_2$ and Nb film; (b) The XRD pattern of TF-$NbSe_2$.

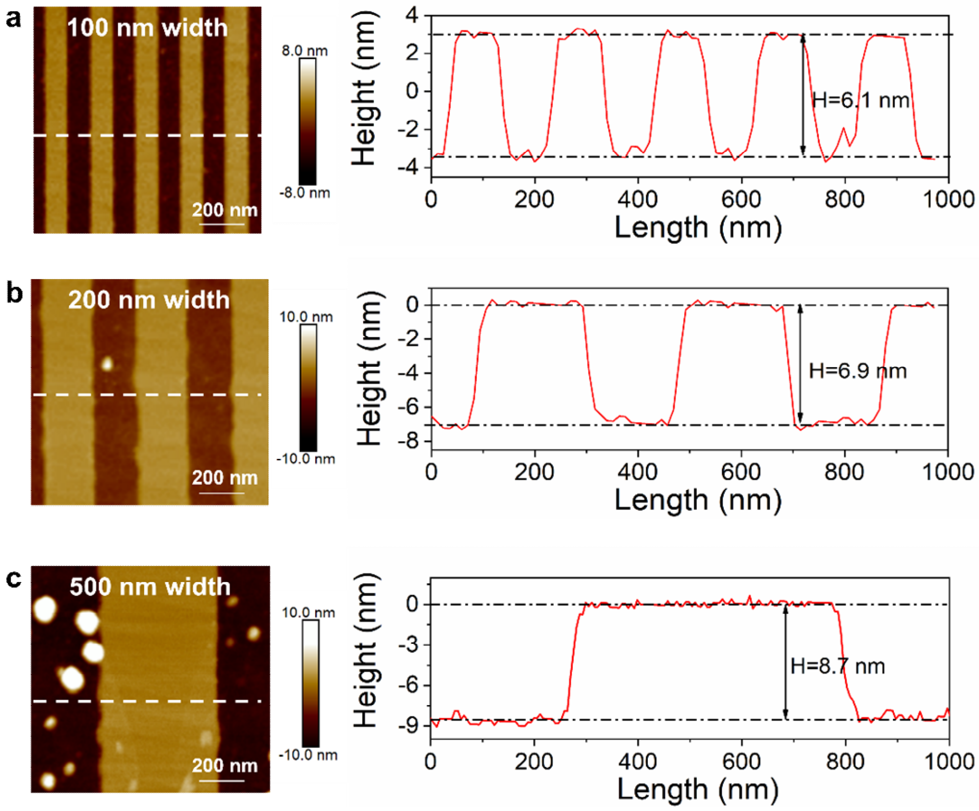

Supplementary Figure 4 Tunability of width and thickness of TF-$NbSe_2$ nanowires. (a) 100 nm width and 6.1 nm height; (b) 200 nm width and 6.9 nm height; (c) 500 nm width and 8.7 nm height.

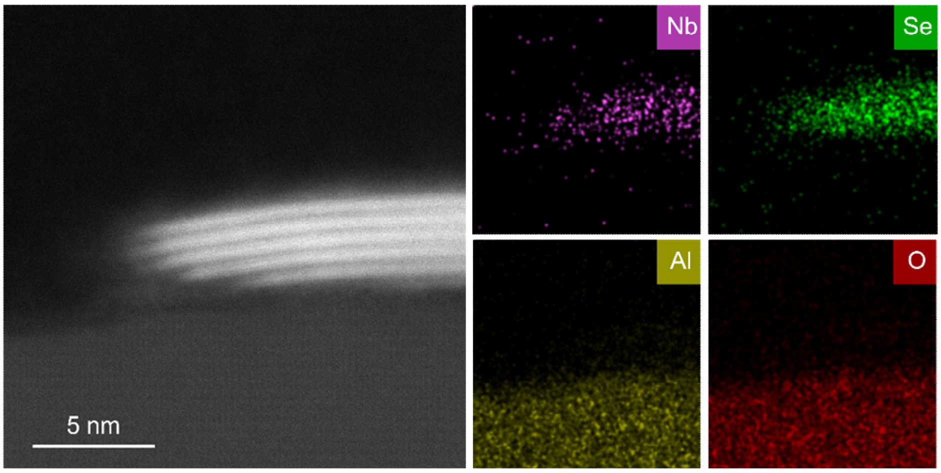

Supplementary Figure 5 Atomic-resolution STEM image of TF-$NbSe_2$ nanowire at the edge and corresponding EDS elemental mappings.

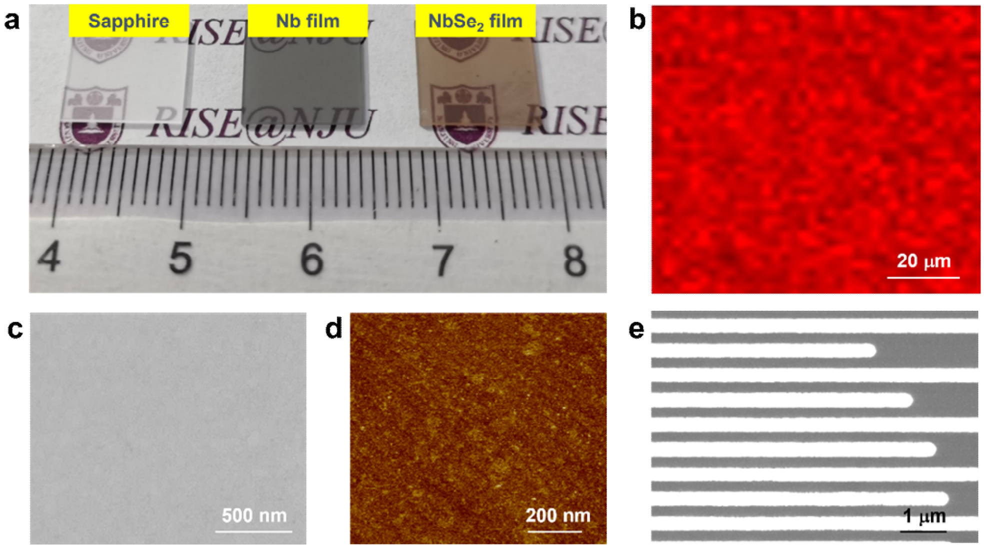

Supplementary Figure 6 Characterization of the TP-$NbSe_2$. (a) Photograph of 1 cm × 1 cm bare sapphire, Nb film, and $NbSe_2$ film. (b) Raman mapping in A_{1g} mode, (c) SEM and (d) AFM images of the $NbSe_2$ film. (e) SEM image of $NbSe_2$ nanowires obtained by the top-down patterning strategy.

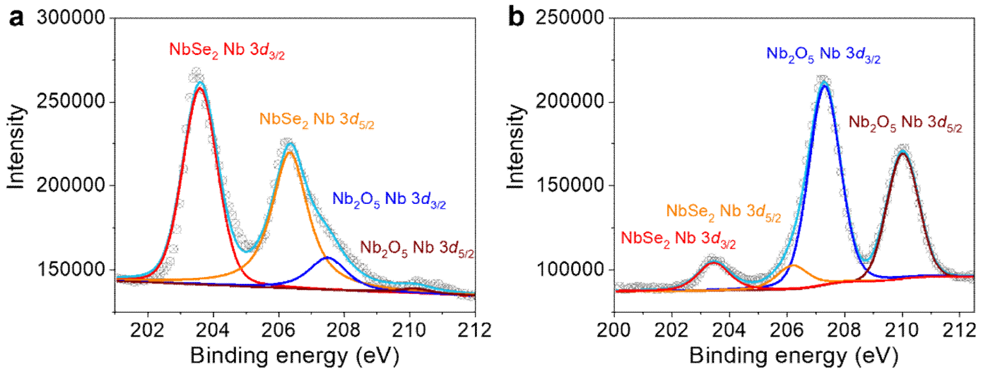

Supplementary Figure 7 Nb 3*d* XPS spectra of (a) TF-$NbSe_2$ and (b) TP-$NbSe_2$.

Supplementary Table 1. The composition analyses of TF-$NbSe_2$ and TP-$NbSe_2$ based on XPS results.

Sample	Area of Nb 3*d* peaks		Composite ratio	Element content (at%)
	$NbSe_2$	Nb_2O_5	$NbSe_2/Nb_2O_5$	Nb/Se/O
TF-$NbSe_2$	323,742.1 (88.1%)	43,767.8 (11.9%)	93.67%/6.33%	1/1.76/0.30
TP-$NbSe_2$	51,501.4 (15.4%)	281,907.7 (84.6%)	26.69%/73.31%	1/0.31/2.12

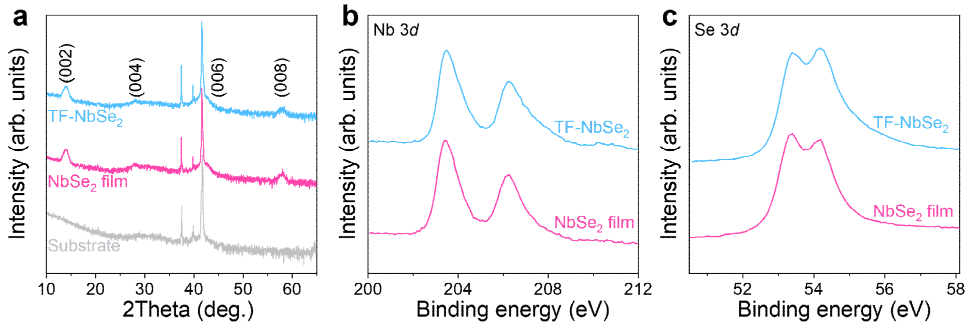

Supplementary Figure 8 (a) XRD patterns, and (b) Nb 3*d* and (c) Se 3*d* XPS spectra of TF-$NbSe_2$ and $NbSe_2$ film.

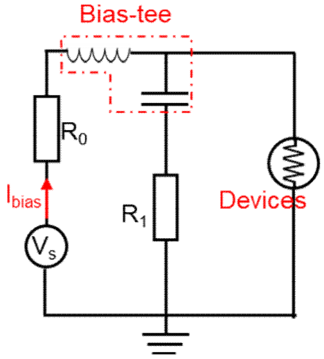

Supplementary Figure 9 Schematic diagram of the measuring circuit of the $NbSe_2$ devices.

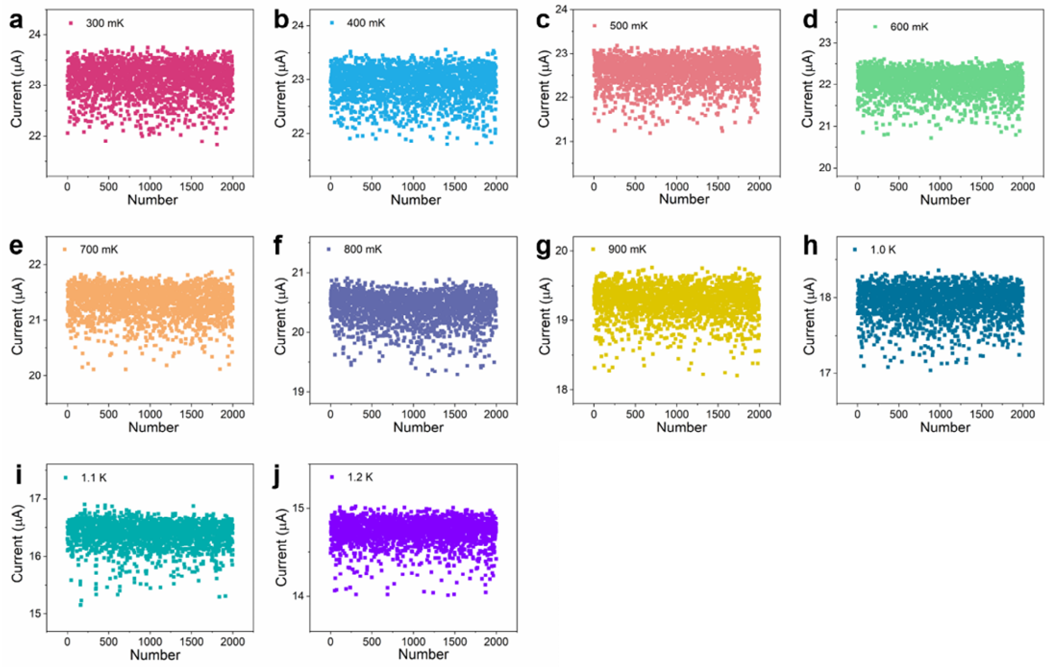

Supplementary Figure 10 Distribution of I_{sw} at different operation temperatures of 200-nm-wide $NbSe_2$ nanowires for 2000 *I*-*V* sweeps with a sweep rate of 5 nA s^{-1}. (a)

300 mK; (b) 400 mK; (c) 500 mK; (d) 600 mK; (e) 700 mK; (f) 800 mK; (g) 900 mK; (h) 1.0 K; (i) 1.1 K; (j) 1.2 K.

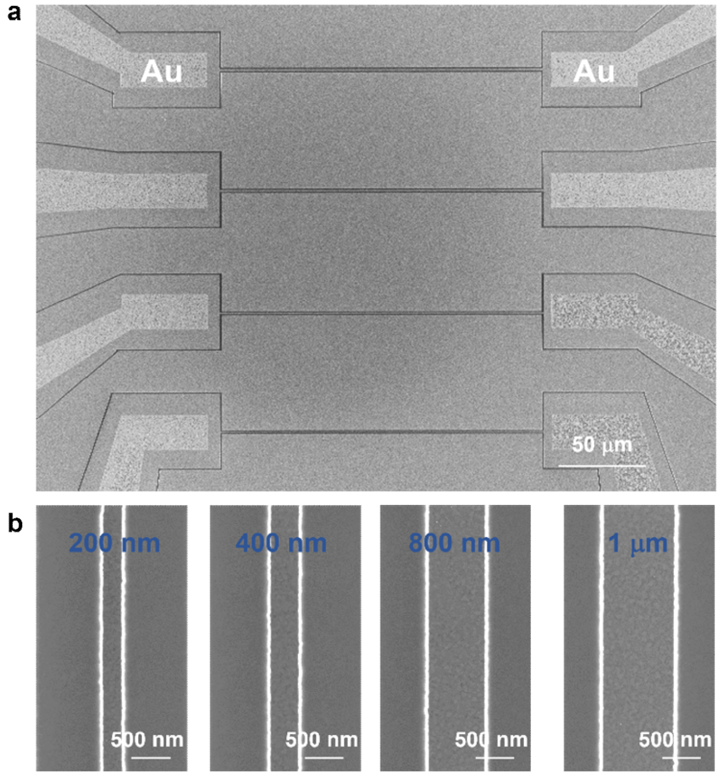

Supplementary Figure 11 SEM images of $NbSe_2$ nanowires. (a) Full image; (b) enlarged images for nanowires with different widths.

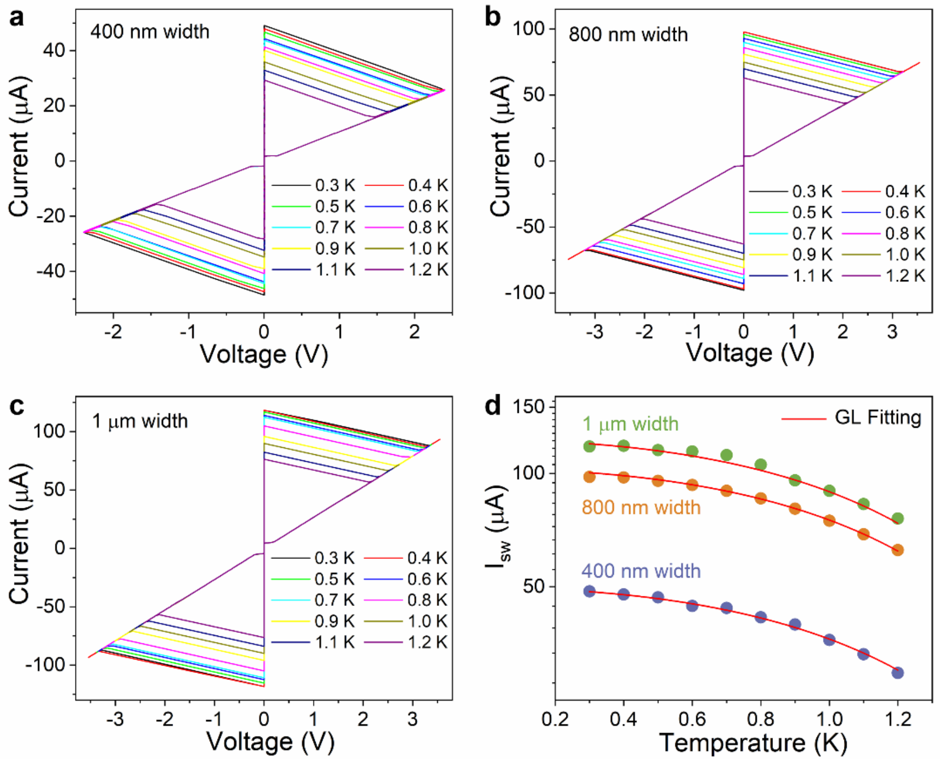

Supplementary Figure 12 *I-V* curves of different linewidth $NbSe_2$ devices under different temperatures. (a) 400 nm; (b) 800 nm; (c) 1 µm. (d) The I_{sw} of different width nanowires under different temperatures fitted by the GL model.

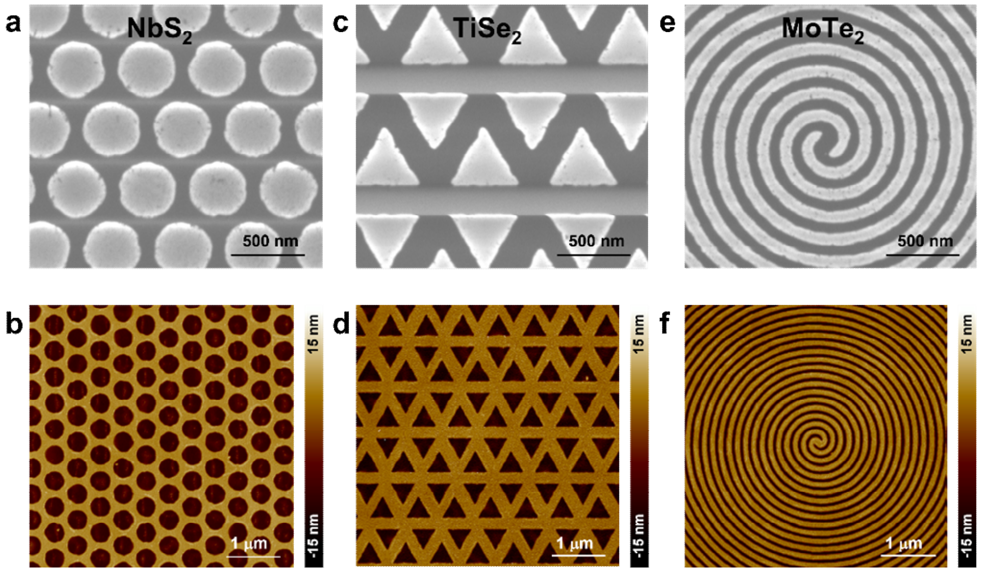

Supplementary Figure 13 SEM and AFM characterizations of NbS_2, $TiSe_2$, and $MoTe_2$ nanopatterns. (a-b) Circle-holey NbS_2; (c-d) triangle-holey $TiSe_2$; (e-f) spiral $MoTe_2$ nanowires.

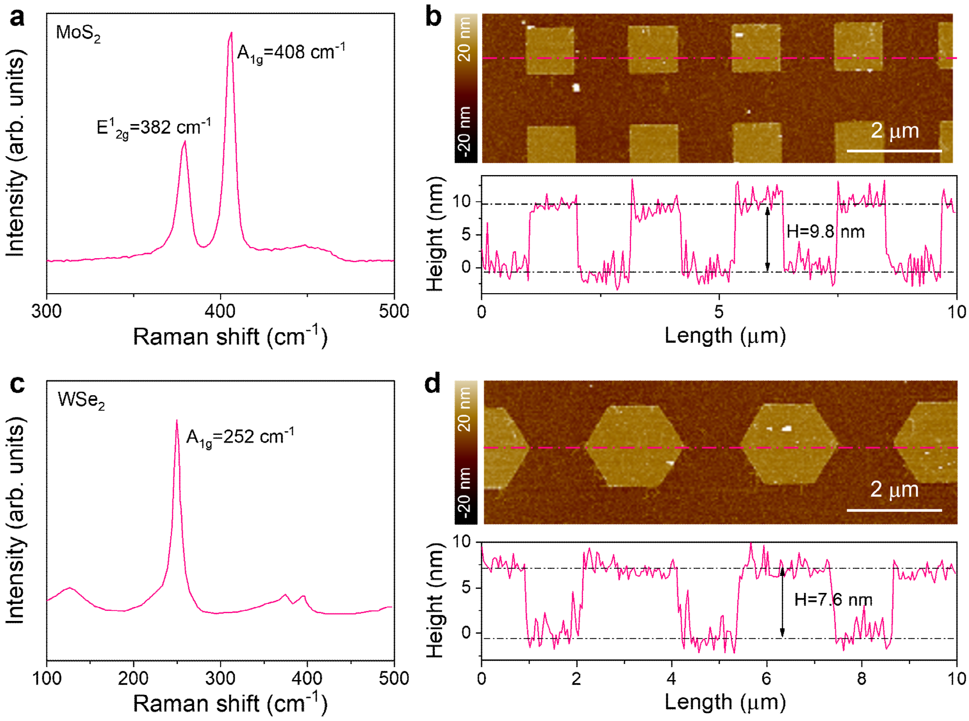

Supplementary Figure 14 Raman spectra and AFM images of MoS_2 square arrays (a-b) and WSe_2 hexagon arrays (c-d).

Supplementary Table 2. Summary of I_{sw}, I_h, and I_{sw}/I_h values for the 200-nm-wide device under different temperatures.

**Temperature (K)**	I_{sw} **(μA)**	I_h **(μA)**	I_{sw}/I_h
0.3	23.618	0.846	27.894
0.4	23.118	0.846	27.307
0.5	22.619	0.846	26.716
0.6	22.120	0.846	26.137
0.7	21.518	0.846	25.423
0.8	20.420	0.846	24.132
0.9	19.622	0.845	23.198
1.0	18.123	0.845	21.429
1.1	16.625	0.845	19.659
1.2	14.728	0.845	17.417

Supplementary Table 3. Specific parameters for fabricating prepatterned metals.

**Fabrication parameters**		**Circle-holey NbS_2**	**Triangle-holey $TiSe_2$**	**Spiral $MoTe_2$ nanowires**
Deposition of metal film	*Vacuum*	< 9×10^{-6} Pa	< 7×10^{-6} Pa	< 2×10^{-5} Pa
	Working power/current	100 W	0.4 A	50 W
	Working pressure	3 mTorr	4 mTorr	1 Pa
	Gas flow rate	80 sccm	100 sccm	23 sccm
	Deposition rate	0.2 nm s^{-1}	0.3 nm s^{-1}	0.15 nm s^{-1}
Patterning of metal film	*Gas*	CF_4	SF_6/CHF_3	CF_4
	Flow rate	40 sccm	40/40 sccm	50 sccm
	Working pressure	4 Pa	4 Pa	5 Pa
	Working power	100 W	100 W	60 W
	Etching time	40 s	29 s	32 s

Supplementary Table 4. Detailed chalcogenization parameters for synthesizing TMDSCs from prepatterned transition metals with different thicknesses.

**TMDSCs**	**Chalcogen Precursors**	**Carrier gas**	**Thickness of prepatterned transition metal**	**Growth temperature**	**Growth time**
$NbSe_2$	Se powder (450 °C)	Ar/H_2 (50/50 sccm)	~4 nm (Nb)	800 °C	10 min
			~3 nm (Nb)	800 °C	8 min
			~2 nm (Nb)	800 °C	6 min
NbS_2	S powder (220 °C)	Ar/H_2 (50/15 sccm)	~3 nm (Nb)	750 °C	10 min

$TiSe_2$	Se powder (450 °C)	Ar/H_2 (50/50 sccm)	~4 nm (Ti)	850 °C	8 min
$MoTe_2$	Te powder (500 °C)	Ar/H_2 (15/15 sccm)	~4 nm (Mo)	600 °C	12 min

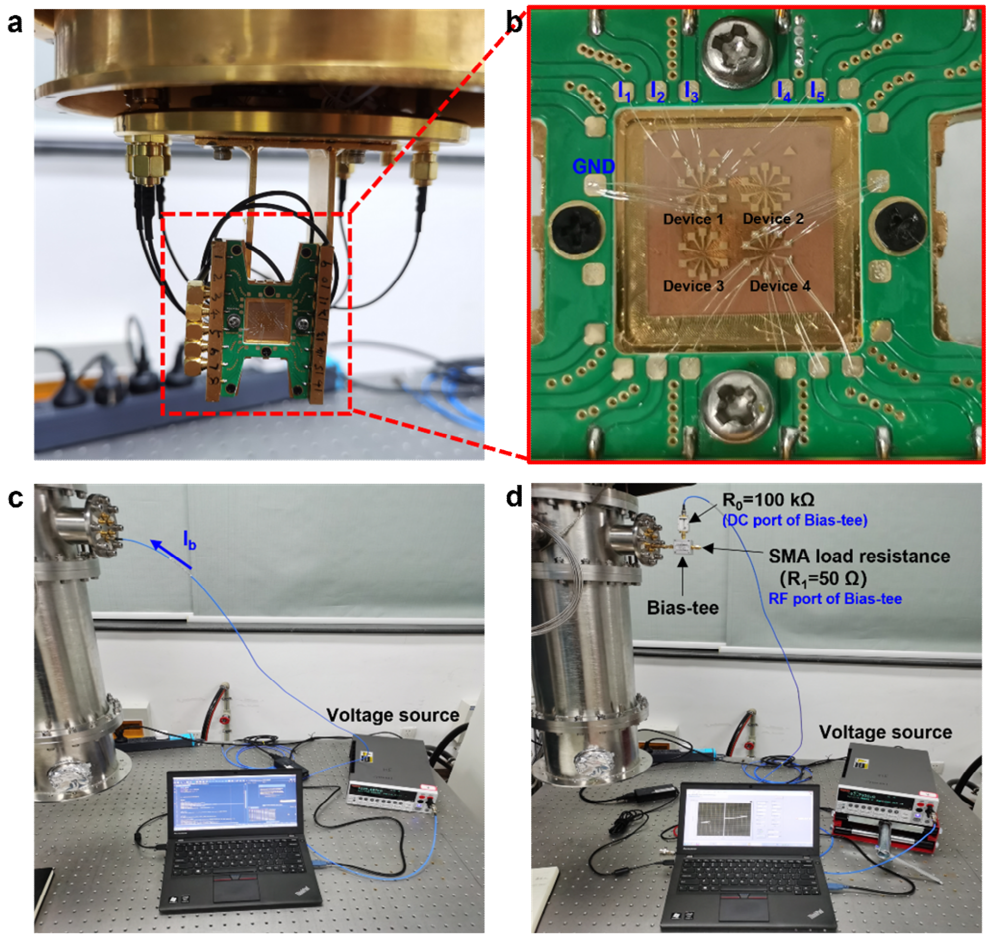

Supplementary Figure 15. Real Pictures for the device measurements. (a) PCB-fixed $NbSe_2$ device connecting to external circuit by low-temperature coaxial cable; (b) enlarged image showing the wire bonding; (c) *R*-*T* and (d) *I*-*V* tests.

The device is set in the refrigerator and connected to an external circuit via a low-temperature coaxial cable, as displayed in **Supplementary Figure 15**a. The TF-$NbSe_2$ device is fixed on the sample holder and connected to the electrodes on the printed circuit board by wire bonding, as shown in **Supplementary Figure 15**b. For the *R*-*T* measurement, Python is used to record the resistance on the source meter (the constant current is set as 1 μA) and the corresponding temperature values (**Supplementary Figure 15**c). For the *I*-*V* test, an isolated voltage source in series with a bias resistor (R_0 = 100 kΩ) was included, which was attached to the DC port of a Bias-tee. The RF port was terminated with a 50 Ω load resistance (R_1), and the DC&RF port was attached to the cryostat feedthrough attached to coax that leads down to the measured device (**Supplementary Figure 15**d). The circuit diagram is also schemed in **Supplementary Figure 9**. The LabView program is used to collect the testing data.
